# Fabrication of tannic acid-incorporated polyvinylpyrrolidone/polyvinyl alcohol composite hydrogel and its application as an adsorbent for copper ion removal

**DOI:** 10.1038/s41598-024-80024-x

**Published:** 2024-11-16

**Authors:** Parichart Chunhakowit, Yada Phabjanda, Atchara Aunwisat, Wutthikrai Busayaporn, Kriangsak Songsrirote, Pornpimol Prayongpan

**Affiliations:** 1 Department of Chemistry, Faculty of Science, Srinakharinwirot University, Bangkok, 10110 Thailand; 2 Synchrotron Light Research Institute, Nakhon Ratchasima, 30000 Thailand

**Keywords:** Tannic acid, Composite hydrogel, Adsorption efficiency, Adsorption mechanism, Copper ions, Environmental sciences, Chemistry

## Abstract

An effective tannic acid-incorporated polyvinylpyrrolidone/polyvinyl alcohol composite hydrogel with high-potential sorption capacity was developed for the removal of copper from aqueous solution. The composite hydrogel exhibited pH-dependent swelling, in which swelling and shrinking occurred reversibly with adjustment of the pH of the medium. At pH 4, the maximal adsorption capacity for copper at 30 °C was 297.0 mg g^-1^, and the adsorbent dose was 4 g L^-1^. The adsorption kinetics were best fitted with a pseudo-second order kinetic model. The adsorption behavior was well predicted by the Freundlish isotherm. The thermodynamics parameters indicated a spontaneous and exothermic reaction with an increase in the entropy of the system. The chemical changes in the film structure before and after adsorption treatment were characterized by Scanning electron microscopy (SEM), Fourier transform infrared (FTIR) spectroscopy, X-ray photoelectron spectroscopy (XPS), and X-ray absorption spectroscopy (XAS). The FTIR, XPS and XAS results confirmed that Cu bound to the oxygens in the -OH, C = O and N-(C = O)- functional groups on the T-HD. XAS analysis revealed the chemical composition and molecular geometry of the adsorbed copper ions. The single-solute adsorption and coadsorption mechanisms, which provide insight into cobalt-copper, nickel-copper, or nickel-cobalt-copper complex solutions, were investigated. The composite hydrogel exhibited excellent regeneration ability in EDTA solution. Notably, the adsorbent retained an adsorption efficiency exceeding 87% even after five regeneration cycles. On the basis of both adsorbent characteristics and adsorption performance, it was determined that the composite hydrogel has the potential to be used as a platform for developing materials to treat wastewater containing high levels of metal contaminants such as those from the electroplating industry.

**Supplementary Information:**

The online version contains supplementary material available at 10.1038/s41598-024-80024-x.

## Introduction

Due to rapid population growth, advances have been made in both industrialization and technology. The industrial sector not only uses available water but also discharges a considerable amount of wastewater, which can be classified according to dominant pollutants such as organic/inorganic compounds. Inorganic contaminants are nonbiodegradable pollutants that persist in the environment. The toxicity of a pollutant is directly proportional to its concentration and the presence of other compounds^1^. Exposure to contaminants such as heavy metals in natural resources, especially natural water sources, has a detrimental effect on the environment^2–4^. The consumption of water containing heavy metals (e.g., Cu(II), Cd(II), and Cr(VI)) that exceed the maximum contaminant limit may negatively affect human health. The permissible limit of copper in industrial effluents was set by the Ministry of Industry of Thailand to 2.0 mg L^-1^, which is the same as the maximum permissible limit of copper ions in drinking water permitted by the World Health Organization (WHO). Many studies have shown that an increase in Cu(II) content in the body leads to serious health problems, such as anemia, vomiting, neutropenia, and growth disorders^5^. Consequently, the discharge of heavy metals from industrial effluents into the environment poses a significant threat to public health and necessitates the implementation of effective methods of detection and remediation strategies.

Electroplating wastewater is a type of industrial effluent that contains high levels (3 − 1500 mg L^-1^) of metal contaminants such as Co, Ni, Cu, and Zn in acidic conditions^6,7^. A variety of techniques, including electrochemical coagulation, chemical precipitation, chemical oxidation and/or reduction, filtration, membrane separation, and evaporation recovery, are being developed to reduce or eliminate heavy metal contamination in water^8,9^. Ni-Cu or Co-Cu cocontaminants are heavy metals that can be present in electroplating wastewater. Ni-Cu contaminants can pose a risk to human health and the environment, as they can cause toxicity, bioaccumulation, and biomagnification^10^. Therefore, it is important to monitor and treat Ni-Cu contaminants in water. Chemical precipitation has become the most widely used method for the removal of metal ions from water. However, this process requires a large amount of chemicals and generates precipitation sludge that needs to be treated^11^. The reported copper concentrations in wastewater range from 2.5 mg L^-1^ to 10,000 mg L^-1^^12^. Another downside of the method is that it is most effective when the wastewater contains a high concentration of metals and is less efficient when the metal content is low.

Adsorption is an effective and uncomplicated process used to remove metal ions from industrial wastewater because of its ease of operation, low cost, and high treatment efficiency. The efficiency of the adsorption process depends on many parameters, including the internal volume, pore size distribution, functional groups, and polarity of the adsorbent as well as the pH of the liquid waste^13–15^. However, most adsorbents are less effective under acidic conditions because the active sites on the adsorbent are not active at low pH^16,17^. In addition, the structural stability of adsorbents decreases at lower pH values, and adsorbed metals are released into the solution, causing secondary pollution^18^. Most adsorption methods require an effluent pH between 6 and 9 to maximize adsorption capacity. As a result, efforts have been made to develop alternative adsorbents that are reusable and effective under a wider range of pH conditions for wastewater treatment.

Hydrogels are three-dimensional network polymers that entrap an abundant amount of water. Hydrogels can be used as a potentially effective candidates for environmental remediation because of their high-water retention capacity and biocompatibility^19–21^. Owning to their distinct properties, such as hydrophilicity, biocompatibility, biodegradability, viscoelasticity, and super absorbency, hydrogel adsorbents can play a prominent role in the capture of heavy metals from contaminated water and can discharge these hazardous pollutants upon changes in the external environment (pH, temperature, etc.)^22,23^. Although this property is beneficial, changing the pH perturbs the adsorption capacity of pH-sensitive hydrogels. The polymer network structure collapses due to electrostatic effects upon phase transition with pH change, which may hinder the regeneration of hydrogels^24^. The development of reinforced composite hydrogels for use as adsorbents under diverse acidic conditions may provide a solution for this pH sensitivity. Elucidating the adsorption mechanism of the hydrogel network structure can provide insights for the design of advanced materials with enhanced adsorption capabilities.

In this work, we produced tannic acid-incorporated polyvinylpyrrolidone/polyvinyl alcohol composite hydrogels (T-HDs) as effective absorbents for the removal of Cu(II) ions in water. The ability of the T-HDs to adsorb Cu(II) in aqueous solution was examined through kinetic and thermodynamic studies under different experimental conditions. The Thermodynamic parameters ∆G, ∆H and ∆S were calculated. The adsorption capacity of the T-HDs for Cu(II) removal in the presence of interfering metal ions (nickel and cobalt) was also determined. Scanning electron microscopy (SEM), Fourier transform infrared spectroscopy (FTIR), X-ray photoelectron spectroscopy (XPS) and X-ray absorption spectroscopy (XAS) were used to investigate the surface and bulk characteristics of the T-HDs before and after copper removal from aqueous solution. Finally, the Cu(II) adsorption mechanism of the T-HDs was discussed. This work presents an advance in the field of water purification, introducing a novel composite hydrogel as an effective adsorbent for copper removal. The findings from this study establish a platform for the application of composite hydrogels in the remediation of wastewater containing high levels of metal contaminants under acidic conditions. Notably, the observed adsorption behavior has not been previously reported, highlighting the innovative nature of this research. This work underscores the potential of composite materials for environmental and chemical applications, a crucial area of research in material chemistry and environmental science.

## Materials and methods

### Materials

Tannic acid (TA, C_76_H_52_O_46_, M_w_ 1701.20) and polyvinylpyrrolidone (PVP, average M_w_ 50 000) were purchased from Sigma-Aldrich. Polyvinyl alcohol (PVA, average M_w_ 85 000 – 124 000) was purchased from ACROS ORGANICS. CuSO_4_⋅5H_2_O reagent, purchased from Carlo Erbo, was used to prepare the CuSO_4_ solution. Sodium acetate and acetic acid were used to prepare buffer solution with a pH of 4. NiSO_4_⋅6H_2_O was purchased from Ajax Finechem. CoSO_4_⋅7H_2_O was purchased from QReC. All other chemicals, including citric acid monohydrate, were purchased from Carlo Erba, and were of ACS grade and used without further purification.

### Preparation of tannic acid-incorporated composite hydrogel

PVA (3 g) was dissolved in 50 mL distilled water and then 3 g PVP and 1.5 g citric acid monohydrate were added to the mixture under constant stirring at room temperature^25^. After that, 2 mL of 0.025, 0.050, and 0.100 M tannic acid solutions (TA) (in 20% ethanol) were slowly added to the above mixture at 0.8, 1.5, and 3.0%, respectively, by volume and stirred at 150 rpm for 3 h at room temperature. The mixture was heated in a 600-W microwave oven for 3 min. After completing the reaction, the solution was cast in a plastic petri dish and dried in an oven overnight at 35 °C for 12 h to obtain 0.9 mm thick tannic acid-incorporated composite hydrogel (T-HD). A Bare hydrogel (HD) was prepared as a control using the previous procedure without adding tannic acid.

**Swelling studies**: The pH-sensitive swelling performance of the hydrogel was determined in buffer solutions of varying pH. Pre-weighed dried hydrogel samples were immersed in a buffer solution (pH 4, 7, and 9) at 30 ± 1 °C for 20 h to swell. The swollen samples were weighed after excess moisture was removed from the surface with filter paper. The swelling ratios, SR, were obtained using the following equation (n = 3).

1 $$SR=\frac{\left({w}_{f}-{w}_{i}\right)}{{w}_{i}}$$

where ${w}_{f}$ and ${w}_{i}$ are the mass (g) of the swollen sample and dried sample, respectively.

**Swelling-deswelling studies**: The swelling-deswelling property was examined using swelling-deswelling cycles. The dried samples were immersed in a pH 4 buffer solution for 20 h and then in a pH 9 buffer solution for 20 h at 30 ± 1 °C. The swelling ratio for each pH after the testing cycle was calculated.

### Adsorption experiments

A set of batch experiments was performed to study the effect of parameters such as solution pH, adsorbent dose, initial concentration, contact time, and temperature on the removal of Cu(II) with T-HD. Batch tests were performed in a sealed conical flask (250 mL) with fixed initial concentrations of Cu(II) solution. The adsorbent dose was kept constant (0.1 g), and agitation time was determined to be 20 h at 120 rpm. Hydrogels (0.1 g) were immersed in DI water before being used as an adsorbent in the batch experiment. At the end of the equilibrium period, supernatants were removed and diluted for UV–visible spectroscopy measurement using a direct method at the wavelength of 788 nm to determine the remaining Cu(II) concentration^26^. All measurements were performed in triplicate.

The calculation of adsorption capacity (q_e_) is based on the amount of Cu(II) adsorbed per unit weight of the T-HD at equilibrium (mg g^-1^) as per the following equation.

2 $${q}_{e}=\frac{\left({C}_{0}-{C}_{e}\right)V}{m}$$

where ${q}_{e}$ (mg g^-1^) is the adsorption capacity, V (L) is the volume of solution, ${C}_{0}$ and ${C}_{e}$ (mg L^-1^) are the initial and equilibrium concentrations of Cu(II), respectively, and $m$ (g) is the mass of dry adsorbent used in the experiments.

**Effect of solution pH:** The effect of pH on Cu(II) adsorption was studied by varying the pH from 1 to 4. The concentration of Cu(II) solution used for this study was 0.2 M in the buffer solution. The pH of the samples was adjusted by adding 0.1 M HCl or 0.1 M NaOH to the prepared solution. The pH of solutions was measured with a pH meter (MP 220 pH Meter; Mettler Toledo). The adsorbent dose used for this study was 0.1 g and the agitation time was 20 h at 120 rpm. Adsorption was not observed at pH ≥ 5 due to Cu(OH)_2_ precipitation.

**Effect of the adsorbent dose:** The effect of the adsorbent dose was performed by varying the amount of T-HD using a fixed solution volume. The experiment was conducted by adding different amounts of T-HD (0.02–0.12 g) in 25 mL of 0.2 M Cu(II) solution at pH 4 (120 rpm, 30 ± 1 °C) until equilibrium time was reached.

**Effect of initial Cu(II) concentration:** The effect of initial concentration was performed by varying the initial concentration of Cu(II) from 0.02 to 0.35 M at pH 4. The experiment was conducted by adding 0.1 g T-HD in 25 mL of different initial concentrations of Cu(II) solution at pH 4 (120 rpm, 30 ± 1 °C) until the equilibrium time was reached.

**Effect of contact time:** The effect of contact time was used to investigate the adsorption kinetic. The adsorption capacity of the adsorbent for Cu(II) ions was determined at different reaction times. The effect of initial concentration on Cu(II) adsorption kinetics were investigated. A set of batch experiments was conducted using 0.5 g of T-HD and 125 mL of 0.05–0.20 M of Cu(II) at pH 4 (120 rpm, 30 ± 1 °C). The 0.5 mL solution was withdrawn at a certain time (0.5, 1, 2, 4, 6, 8, 10, and 12 h) to determine the concentration of Cu(II) ion in the remaining solution by absorbance measurements performed using a microplate reader BioTEk SYNERGY HTX. One hundred microliters withdrawn solutions were applied in triplicate in each well and measured at 788 nm at 30 ± 1 °C. The average absorbance was used for further evaluation. The adsorption capacity of adsorbing Cu(II) ions at time t, q_t_ (mg g^-1^), was calculated using the following equation.

3 $${q}_{t}=\frac{\left({C}_{0}-{C}_{t}\right)V}{m}$$

where ${C}_{0}$ and ${C}_{t}$ (mg L^-1^) are the initial and remaining concentrations of Cu(II) at time t, respectively, V (L) is the volume of Cu(II), and $m$ (g) is the mass of dry adsorbent used in the experiments.

**Effect of temperature:** The effect of temperature was used to investigate the thermodynamics of the adsorption process. The temperature was held at four different levels (10, 20, 30, and 40 °C) with a fixed initial concentration of Cu(II) solution (25 mL, 0.2 M) at pH 4. The adsorbent dose used for this study was 0.1 g and the agitation time was 20 h at 120 rpm.

**Effect of interfering ions:** The effect of interfering ions was used to investigate the selectivity of T-HD for Cu(II) ions adsorption and to predict the adsorption mechanism of the copper on T-HD adsorbent in the presence of competing metal ions. The experiment was conducted using sample solutions containing 0.1 M Cu(II) and 0.1 M interfering ions at pH 4. The adsorbent dose used for this study was 0.1 g in 25 mL of mixed metal ions solution and agitation time was 20 h at 120 rpm. The remaining copper in mixed solution was investigated by UV-visible spectroscopy measurement using Vierordt’s method^27^.

### Regeneration experiments

The regeneration experiment was conducted to determine the cycle efficiency of T-HD adsorbent. For this experiment, the T-HD was first immersed in 25 mL of 0.2 M Cu(II) solution for 20 h. Desorption was done by transferring the T-HD saturated with Cu(II) to 20 mL of 0.1 M EDTA solution and shaking in a sonicating bath for 15 min at 30 ± 1°C^28^. The desorbed T-HD was washed with 100 mL deionized water, dried in the oven at 35 °C, and reused as an adsorbent in the next adsorption–desorption cycle. The adsorption experiment was repeated four more times. The adsorption efficiency is the ratio between the second and first adsorption capacity as per the following equation.

4 $$\text{Cycle efficiency }= \left(\frac{{q}_{n}}{{q}_{1}}\right)\times 100\%$$

where ${q}_{n}$ and ${q}_{1}$ are the n^th^ and first adsorption capacity of T-HD adsorbent, respectively.

### Characterizations

T-HD samples before and after adsorption tests were freeze-died for 48 h before being analyzed. The surface morphology was measured using scanning electron microscopy (SEM) (JEOL, JSM-6510LV, Japan) at 5000 × at an acceleration voltage of 10 kV. The Brunauer-Emmett-Teller (BET) model was adopted for specific surface area analysis though nitrogen adsorption isotherm at 77 K by using automatic adsorption system (Quantachrome instrument). The samples were degassed at 180 °C for 6 h prior to measurement. The X-ray photoelectron spectroscopy (XPS) measurement was performed using a Kratos Shimadzu Axis Ultra DLD machine (Kratos, Manchester) equipped with the Al K_α_ radiation. All the binding energies were calibrated to the neutral C1s peak at 284.6 eV to compensate for the surface charging effects. The Fourier transform infrared (FTIR) spectroscopy was determined using a Spectrum Two PerkinElmer FTIR spectrometer within 1000–4000 cm^-1^. UV–visible spectroscopy measurements were carried out using a V-750 JASCO spectrophotometer.

Spectra of X-ray absorption near-edge structure (XANES) and extended X-ray absorption fine structure (EXAFS) were measured at Beamline 8, Synchrotron Light Research Institute (SLRI), Nakhon Ratchasima, Thailand. In the experiment, X-ray generated by a 1.2 GeV storage ring, operating at a beam current ranging from 50 to 150 mA, was monochromated using a Ge(220) double-crystal monochromator. The Co, Ni, and Cu K-edges XANES and EXAFS spectra of references and samples were collected in transmission and fluorescence modes at room temperature, respectively. In the transmission mode, in-house ionization chambers were used to collect I_0_ and I_1_ currents while in the fluorescence mode, 13-element silicon drift detector was used to collect the fluorescence signal from the samples. CoSO_4_⋅7H_2_O, NiSO_4_⋅6H_2_O, and CuSO_4_⋅5H_2_O were measured for Co, Ni, and Cu K-edges XANES spectra as references. To prepare the references, the compounds were smeared as a thin layer between kapton tapes. For samples, one side of kapton tape was replaced by a piece of polyimide tape (Lamar Inc, USA). After that, all spectra were analyzed using Athena software^29^, the pre-edge and post-edge background were selected and subtracted to normalize XANES. The spectra of all edges were calibrated by defining the first derivative peaks of Co, Ni, and Cu metal foils. To determine the local structure around Co, Ni, and Cu, EXAFS data were fitted with the trial atomic models using Artemis software^29^.

### Data analysis

All measurements in this study were conducted in triplicate, and the mean results (+/- SD) are presented. Microsoft EXCEL software was used to solve the adsorption isotherms and kinetic models.

## Results and discussion

### Swelling properties and swelling-deswelling cycles

**Fig. 1:**
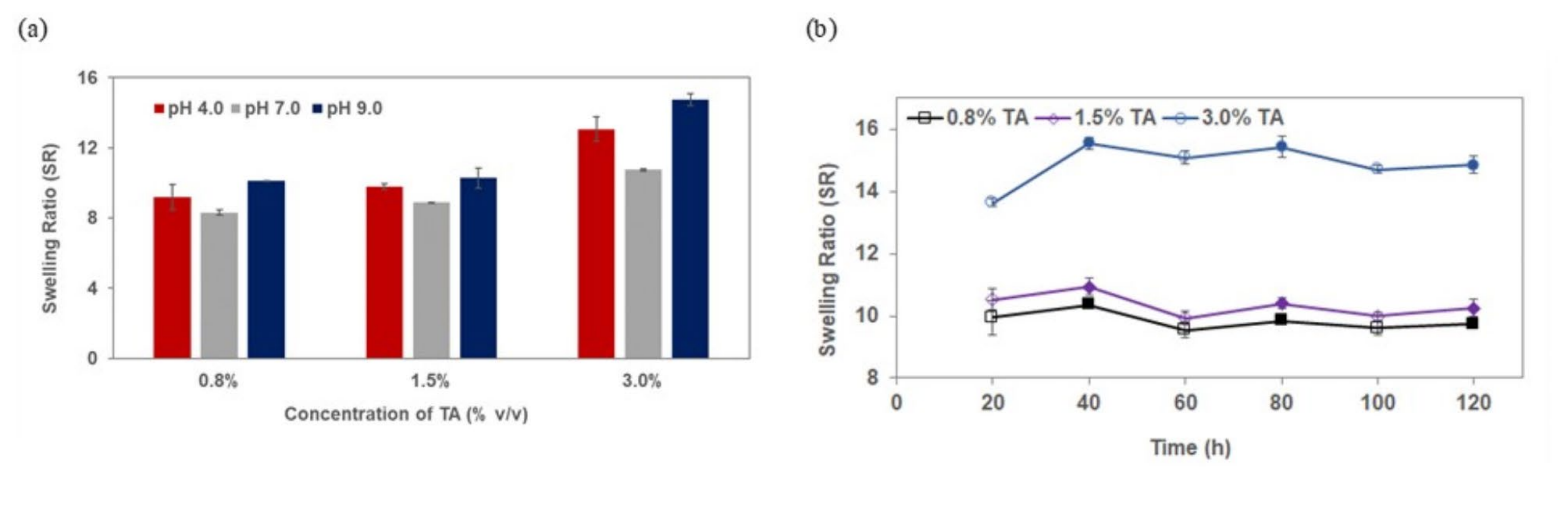
(**a**) The swelling ratio of T-HD in an aqueous buffer solution pH 4, pH 7, and pH 9. (Mass of T-HD = 0.3 g, buffer solution volume = 40.00 mL; swell time = 20 h) (**b**) The reversible changes in the swelling ratio of T-HD containing different amount of TA (0.8%, 1.5%, and 3.0%) between pH 4 (☐◇○) and pH 9 (■⬥●).

Equilibrium swelling is an important consideration in hydrogel efficiency for adsorption applications. The higher the swelling ratio, the faster the adsorbates can diffuse into the hydrogel network^30^. Figure 1a. shows a graph of the swelling ratio of T-HD as a function of pH. The effect of the amount of tannic acid on the swelling ratio (SR) of the hydrogels in a buffer solution of pH 4, 7, and 9 were determined. The results showed that the swelling ratio of T-HD increased with the concentration of TA in T-HD from 0.8 to 3.0%. This might have resulted from the higher TA content that improved the hydrogen bond interactions between the TA and polymer matrix^31^, consequently enhancing the swelling property of the hydrogel. However, when more than 3% of TA was used during the preparation process of the hydrogels, a partial TA release occurred in the aqueous solution possibly due to the excessive amount of TA required in creating cross-linked products^32^.

According to the SR of the T-HD in aqueous solution with varied pH (Fig. 1a.), it was found that the hydrogels were pH sensitive. The SR of T-HD showed the greatest swelling ability at pH 9 (pH 9 > pH 4 > pH 7). The lower SR was observed under acidic pH (pH 4) because most of the phenolic hydroxyl groups were protonated, leading to the hydrogen bond formation between the phenolic hydroxyl groups. The highest SR was found under basic pH (pH 9) because most of the phenolic hydroxyl groups were ionized, leading to the electrostatic repulsion that caused the extension of polymer chains. The lowest SR observed at pH 7 may be due to a lower degree of ionization of phenolic groups. Thus, pH-sensitive T-HD acted as an effective adsorbent under a certain pH.

The swelling property after three cycles of swelling and deswelling was also investigated. Figure 1b. shows the reversible changes in the SR of the hydrogels between pH 4 and pH 9 at 30 °C. The hydrogel shrunk at pH 4 and swollen at pH 9. During the first swelling-deswelling cycle for T-HD containing 3.0% TA, the swelling ratio increased from 13.7 to 15.6 after the change in pH of the swelling medium from pH 4 to pH 9. The T-HD shrunk again at pH 4 to a swelling ratio value of 15.0. During the second cycle, the swelling ratio of the T-HD increased again at pH 9, and then decreased at pH 4. The equilibrium swelling ratio of the hydrogel was similar at the same pH at the second cycle and decreased after the third cycle. The data indicated that the swelling-deswelling behavior of hydrogels was reversible when changing the pH of the medium. This could indicate that the structure of T-HD can withstand the movement or relaxation of the polymer chains reversibly^33^.

### Adsorption experiments

The experimental conditions and concentrations of pollutants play a crucial role in the adsorption efficiency. For the adsorption experiment, T-HD containing 3.0% of tannic acid solution was selected for the Cu(II) adsorption study. Parameters, including solution pH, adsorbate initial concentration, contact time, temperature, and interfering ions, that could affect the removal of copper from aqueous solution were investigated.

**Effect of pH:** Medium acidity is a significant factor in controlling the adsorption behavior of a cationic molecule onto an adsorbent as it may affect the adsorbent surface charge. To understand the presence of the functional groups on the adsorbent surface, the point zero charge was determined in the pH range of 2.0–12.0^34^. The results are presented in Fig. S1. The pH of the point of zero charge (pH_pzc_) was found to be 3.0. At this point, there was no surface charge to be neutralized by ions^35,36^. At pH values below pH_pzc_, T-HD surface became positive, creating electrostatic repulsion between the surface and the metal cations and leading to the depletion of the cation adsorption. Conversely, at pH values greater than pH_pzc_, the T-HD surface became negatively charged. This negative charge generated an electrostatic attraction between the negatively charged surface and the metal ions, enhancing the cation adsorption capacity.

**Fig. 2:**
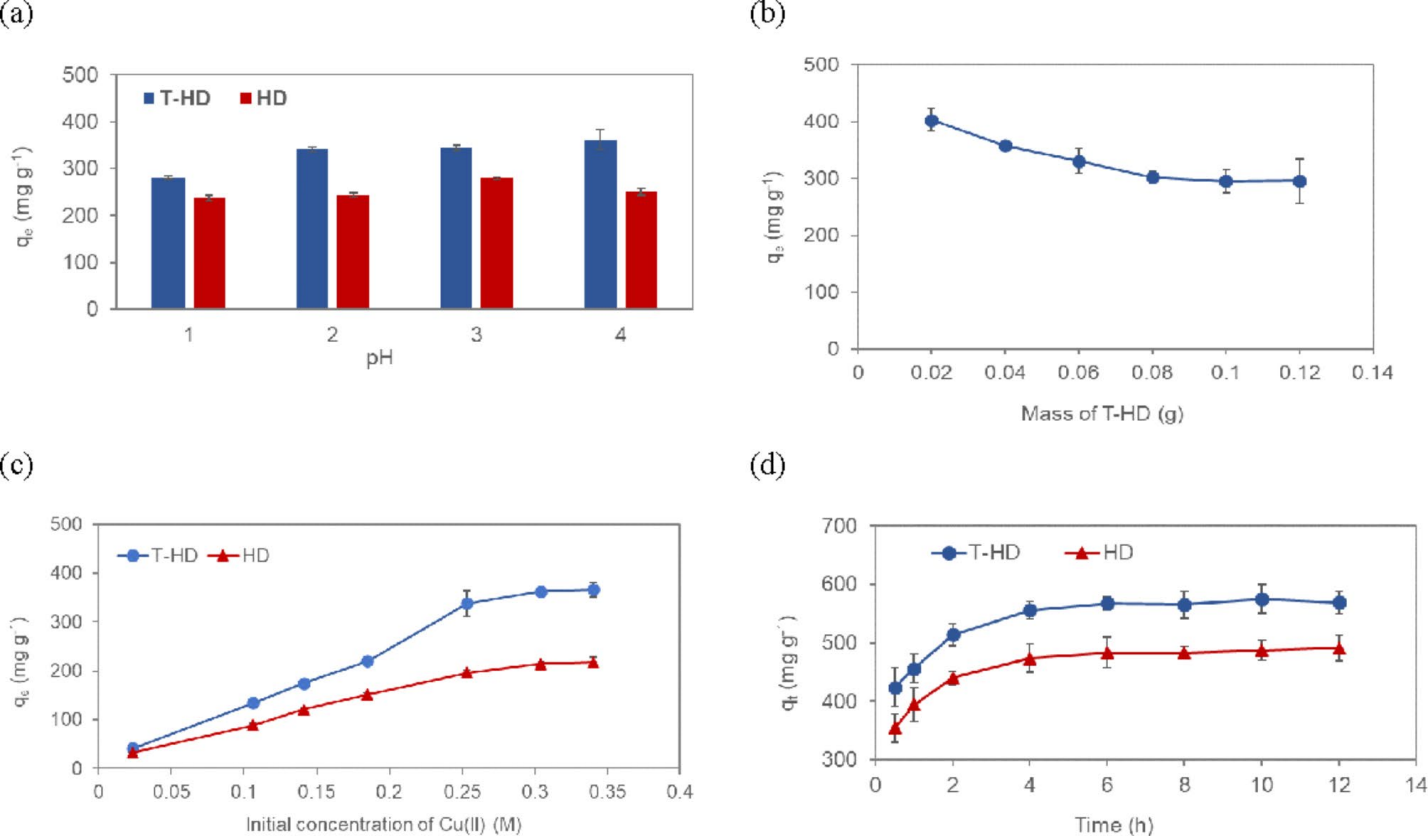
(**a**) Effect of pH (initial concentrations 0.2 M, contact time 20 h, temperature 30 °C). (**b**) Effect of adsorbent dose (n = 3) (initial concentrations 0.2 M, contact time 20 h, temperature 30 °C). (**c**) Effect of initial concentration (adsorbent dose 4 g L^-1^, contact time 20 h, temperature 30 °C). (**d**) Effect of contact time (n = 3) (adsorbent dose 4 g L^-1^, temperature 30 °C) on the removal of Cu(II) by T-HD.

The effect of pH on Cu(II) adsorption was determined at different values from 1 to 4, and the results are shown in Fig. 2a. The equilibrium adsorption capacity (q_e_) of the T-HD is influenced by solution pH. The result showed that the adsorption capacity increased with increasing pH and peaked at pH 4. At pH values higher than 4, the precipitate of Cu(OH)_2_ was formed and affected Cu(II) adsorption. At lower pH values, the adsorption capacity was low. This may be attributed to the reduction of the number of active sites for Cu(II) adsorption caused by the existence of more hydrogen ions, which competed with the metal cations. As pH increased, more active sites were available for Cu(II) adsorption due to a decrease in the amount of hydrogen ions^37^. The higher copper adsorption capacity was observed in T-HD adsorbent compared to HD adsorbent at pH 1 to 4. The results indicated that T-HD was most effective for Cu(II) adsorption in water at pH 4, and electrostatic attraction occurred between Cu(II) ions and the negatively charged adsorbent. Herein, an optimal working pH value of 4 was selected for further adsorption studies.

**Effect of adsorbent dose:** The adsorption dosage is an important factor in determining the adsorbent capacity for a given initial concentration of Cu(II) solution. Figure 2b. presents the effect of T-HD dosage on the adsorption of copper. It was found that the adsorption capacity for Cu(II) adsorption onto T-HD decreased as the T-HD dosage increased due to the availability of surface area of the T-HD. T-HD of 0.1 g was used as the optimal amount of adsorbent for the removal of Cu(II).

**Effect of initial Cu(II) concentration:** Initial concentration played an important role in the adsorption capacity of Cu(II) on T-HD adsorbent. The adsorption capacity of adsorbent for Cu(II) was determined at different initial concentrations (Fig. 2c). The adsorption capacity increased and remained stable as the concentration increased. This is likely attributed to an increase in the driving force of concentration gradient when the initial concentration increased. From the study of various adsorption parameters, the experimental results indicated that at an adsorbent dose of 4 g L^-1^ and initial concentration of 0.2 M, the adsorption capacity of T-HD and HD were 297.0 mg g^-1^ and 176.5 mg g^-1^ at equilibrium time.

**Effect of contact time:** The kinetics of the adsorption was studied to investigate how fast the adsorption process reached equilibrium and to determine the contact time needed to achieve the highest adsorption capacity. Figure 2d. shows the effect of contact time to the adsorption capacity of Cu(II) onto T-HD and HD with 0.20 M initial concentration and 4 g L^-1^ dose of adsorbent. The adsorption initially increased rapidly and then slowed down when the contact time was greater than two hours. The results can be ascribed to more vacant surface sites were available for Cu(II) adsorption during the initial stage, and then, fewer vacant surface sites were available for the copper ions due to repulsive forces between the copper particles on the T-HD and the bulk phase. Our study showed that Cu(II) was adsorbed by and reached equilibrium on the T-HD film after 6 h. The maximum adsorption capacity of T-HD was greater than that of HD, introducing that T-HD was a better adsorbent. The effect of initial concentration on Cu(II) adsorption behavior onto T-HD was studied at different initial Cu(II) concentrations of 0.05, 0.10, 0.15, and 0.20 M with the adsorbent dose 4 g L^-1^. The results showed that the adsorption process was initially fast and then slowed down as it approached the sorption equilibrium state for all initial Cu(II) concentrations (Fig. S2.). By increasing the initial adsorbate concentration, the adsorption capacity increased as governed by the driving force for mass transfer in the bulk and surface layer. The adsorption capacity depended on its initial concentration and higher concentration increased the overall mass transfer which enhanced the metal adsorption onto the adsorbent surface^38^.

### Adsorption kinetics

To investigate the adsorption mechanism and adsorption characteristics, three different kinetic models; pseudo-first order model, pseudo-second order model, and intraparticle diffusion model (IPD), were used to interpret the results obtained from the study of the effect of contact time^39^.

The pseudo-first order kinetic model postulated that the rate of adsorption is proportional to the number of active sites available on an adsorbent^40,41^. The pseudo-first order rate equation is expressed as follows.

5 $$ln \left({q}_{e}-{q}_{t}\right)=ln \left({q}_{e}\right)-{k}_{1}t$$

where q_t_ and q_e_ (mg g^-1^) are the amount of Cu(II) adsorbed at time t and at equilibrium time, respectively. Parameter k_1_ (min^-1^) is the adsorption rate constant obtained from the plot of ln (q_e_ - q_t_) versus time (t).

The pseudo-second order kinetic model assumes that the rate-limiting step of adsorption involves either electron sharing or electron transfer between adsorbate and adsorbent^42^. The experimental data from the batch experiments studied on the effect of contact time was also evaluated according to the pseudo-second order kinetic model expressed as follows.

6 $$\frac{t}{{q}_{t}}=\frac{1}{{k}_{2}{q}_{e}^{2}}+\frac{t}{{q}_{e}}$$

where k_2_ (g mg^-1^ h^-1^) is the adsorption rate constant calculated from a plot between t/q_t_ against time (t).

Moreover, adsorption models such as intraparticle diffusion model or IPD model were used to further analyze the adsorption process, calculated as follows^43^.

7 $${q}_{t}={k}_{id}{t}^{0.5}+C$$

where q_t_ (mg g^-1^) is the adsorption capacity at time t. Parameter k_*id*_ (mg g^-1^ min^0^^.5^) is the IPD rate constant of intraparticle diffusion. C (mg g^-1^) is the model parameter related to the thickness of boundary layer on diffusion.

**Fig. 3:**
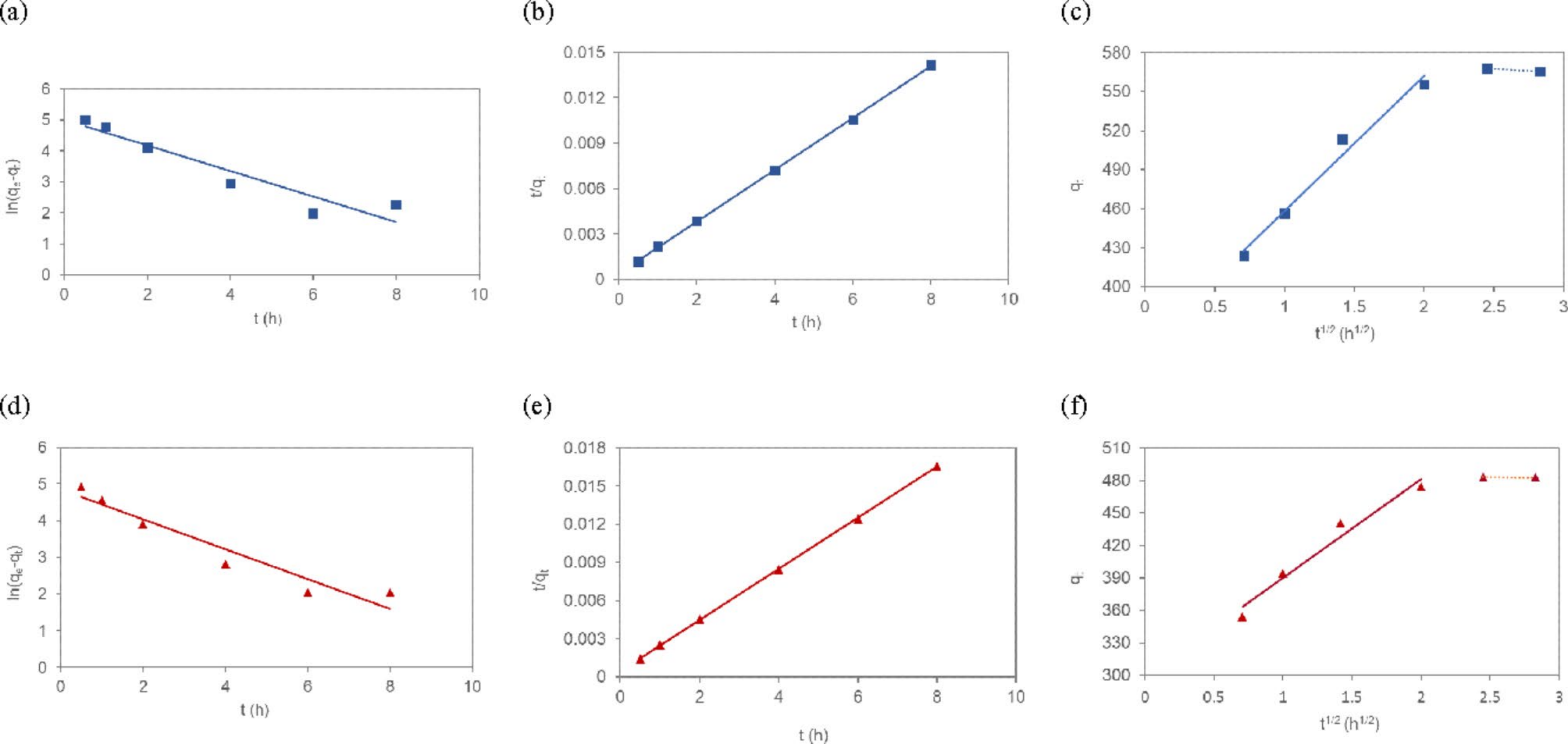
(**a**) pseudo-first order (**b**) pseudo-second order and (**c**) intraparticle diffusion kinetic models on the T-HD (n = 3) and (**d**) pseudo-first order (**e**) pseudo-second order and (**f**) intraparticle diffusion kinetic models on the HD (n = 3) for the adsorption of Cu(II).

Pseudo-first order, pseudo-second order, and IPD models were applied to fit the experimental data for T-HD (Fig. 3a to c) and HD adsorbents (Fig. 3d to f). The kinetic parameters associated with the models and adsorption rate constant of Cu(II) are shown in Table 1. Overall, the linear regression correlation coefficient value (R^2^) obtained from the pseudo-second order kinetics is close to 1.0 (0.999). The calculated equilibrium adsorption value, q_e_ (cal), derived from the pseudo-second order kinetic model for Cu(II) adsorption is 585 mg g^-1^_._ Due to the difference between q_e_ (cal) and q_e_ (exp) values and the higher linear regression correlation coefficient value (R^2^), the adsorption of Cu(II) on T-HD is best described by the pseudo-second order kinetic model. This implies that the pseudo-second order model was predominant for this adsorbent system in the adsorption process over a prolonged period. The rate of adsorption was largely attributed to the adsorption capacity rather than the adsorbate concentration^44^. Similar adsorption behaviors were observed between T-HD and HD for Cu(II) adsorption. However, the pseudo second rate constant, k_2_ as a time-scale factor, was lower for T-HD than for HD suggesting a longer time was required to reach adsorption equilibrium. This observation suggests that the transport of adsorbate through the pores to the adsorption sites might be influenced by the pore geometry of the adsorbents^45^.

**Table 1:** Kinetic parameters of the adsorption of Cu(II) on the T-HD and HD.

Adsorbent	Pseudo-first order			Pseudo-second order			Intraparticle diffusion model		
	R^2^	k_1_ (h^-1^)	q_e_ (mg g^-1^)	R^2^	k_2_ (g mg^-1^ h^-1^)	q_e_ (mg g^-1^)	R^2^	k_i_ (mg g^-1^ h^-1/2^)	C (mg g^--1^)
T-HD	0.899	0.413	149	0.999	0.00721	585	0.980	103	355
HD	0.924	0.406	127	0.999	0.00854	498	0.963	91.5	299

The kinetic data were further investigated by IPD model to explore the diffusion mechanism for Cu(II) adsorption onto T-HD and HD. The plots between q_t_ versus t^1/2^ (Fig. 3c and f) were divided into two linear parts: the sharp rise portions and a plateau portion. Moreover, the deviation of the two linear lines from the origin in the intraparticle diffusion plot indicates that both intraparticle diffusion and external diffusion contribute to the adsorption process. In the first stage, Cu(II) diffused from the solution to the outer surface of T-HD at a higher rate. In the second stage, Cu(II) ions continued to diffuse into the interior pores of the T-HD and were adsorbed on the porous surface at a lower rate. The internal structure of the adsorbent became smaller as Cu(II) began to diffuse into the inner surface of the adsorbent. As a result, the free path of the particles in the pores was reduced, effectively increasing the absorption rate^46^. Based on the calculated parameters listed in Table 1, the intraparticle diffusion rate constant (k_i_) in the first regression line for the adsorption of Cu(II) onto T-HD increased compared to HD which indicated a higher surface adsorption reaction with an active site. Moreover, the larger value of the intercept, C, representing the boundary layer thickness, for T-HD demonstrated that pore diffusion was primary involved in the adsorption process on T-HD.

### Effect of temperature and adsorption equilibrium

**Fig. 4:**
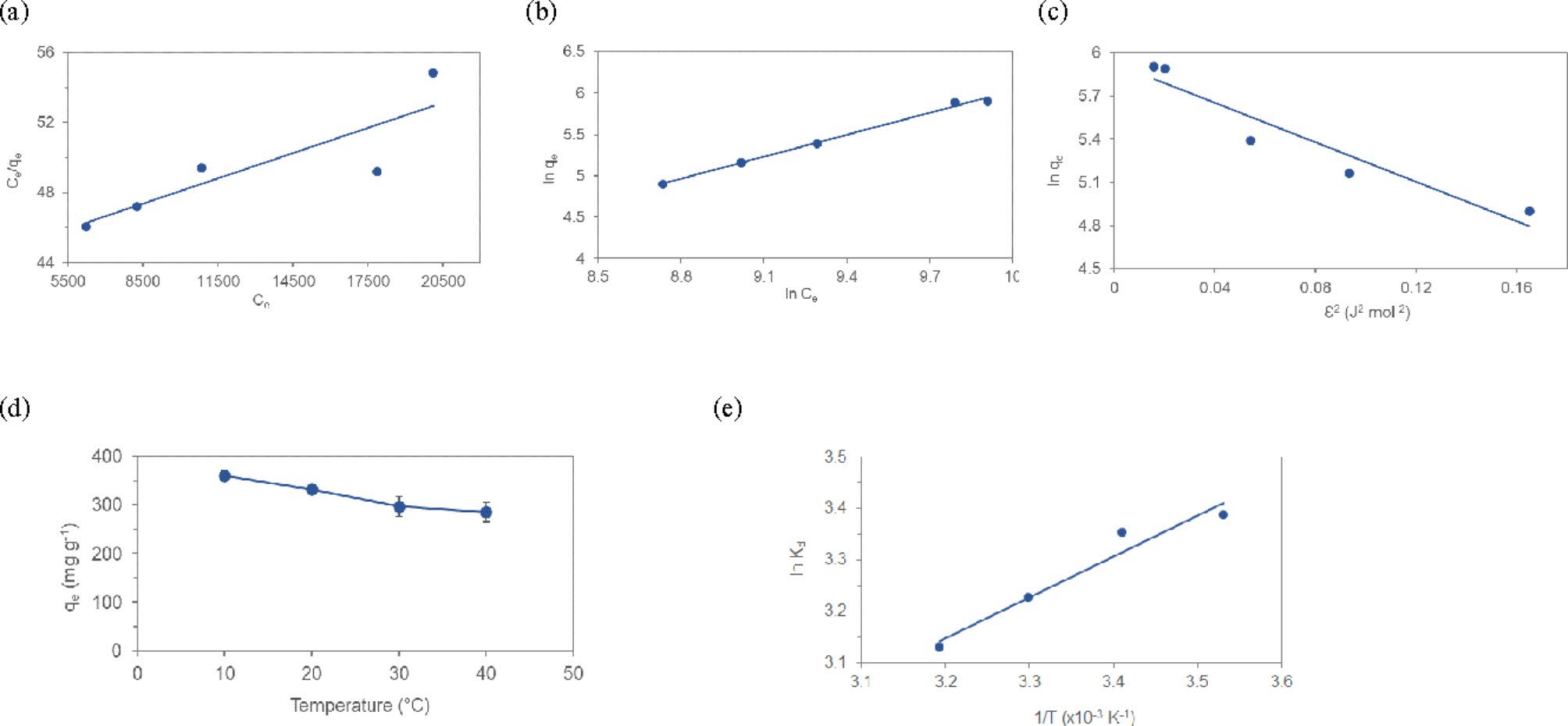
(**a**) Langmuir (**b**) Freundlich and (**c**) Dubinin-Radushkevich isotherms for the T-HD. (**d**) Effect of temperature on adsorption of Cu(II) by T-HD (contact time 20 h, pH 4, adsorbent dose 4 g L^-1^). (**e**) Plot of $ln{K}_{d}$ versus $\frac{1}{T}$ .

To understand how the adsorbate interacts with adsorbents, the adsorption isotherms, Langmuir, Freundlich, and Dubinin-Radushkevich (D-R), were used to model the adsorption data obtained at the equilibrium state (Fig. 4). The Langmuir isotherm models the monolayer on a homogeneous surface with a fixed number of identical sites^47^. This model assumes that the adsorption takes place at a specific adsorption surface and can be expressed by the following linear equation.

8 $$\frac{{c}_{e}}{{q}_{e}}=\frac{1}{{K}_{L}{q}_{m}}+\frac{{c}_{e}}{{q}_{m}}$$

where, ${q}_{e}$ (mg g^-1^) is the adsorption capacity or the amount of Cu(II) per unit of adsorbent, ${q}_{m}$ (mg g^-1^) is the monolayer adsorption capacity, ${c}_{e}$ (mg L^-1^) is the equilibrium concentration of the solution and ${K}_{L}$ (L mg^-1^) is the constant of Langmuir isotherm model, giving information on the binding energy of adsorption process.

Freundlich isotherm models the multilayers on heterogeneous surfaces^48,49^. This isotherm can be defined by the following linear equation.

9 $$ln {q}_{e}=ln {K}_{F}+\frac{1}{n} ln {c}_{e}$$

where, ${q}_{e}$ (mg g^-1^) is the adsorption capacity, ${K}_{F}$ is the constant of Freundlich isotherm model (mg g^-1^), and $\frac{1}{n}$ is the adsorption intensity and ${c}_{e}$ (mg L^-1^) is the equilibrium concentration of the solution.

The D-R isotherm model describes the adsorption by microporous structure and assumes the distribution of pores in adsorbents to follow Gaussian energy distribution onto heterogeneous surface^50,51^. The D-R isotherm is expressed as follows.

10 $$ln {q}_{e}=ln {q}_{m}-{K}_{D}{\varepsilon }^{2}$$

where, q_e_ (mg g^-1^) is the amount of Cu(II) adsorbed per unit weight of adsorbent, q_m_ (mg g^-1^) is the D-R adsorption capacity, K_D_ (mol^2^ kJ^-2^) is the constant related to mean adsorption energy, determining from the slope by plotting $ln{ q}_{e}$ with ${\varepsilon }^{2}$, and $\varepsilon$ (J mol^-1^) is the Polanyi potential which can be determined as follows.

11 $$\varepsilon =RTln \left(1+\frac{1}{{C}_{e}}\right)$$

where R is the gas constant, T (K) is the solution temperature, ${C}_{e}$ is the concentration of adsorbate at the equilibrium in solution after adsorption (mg L^-1^). The D-R models can be used to determine the mean adsorption energy as follows.

12 $$E=\frac{1}{\sqrt{2{K}_{D}}}$$

It was found that the mean adsorption energy (0.270 kJ mol^-1^) for Cu(II) adsorption on T-HD was smaller than 8 kJ mol^-1^ revealing the predominant on the physical adsorption process^52^. Therefore the adsorption behavior of Cu(II) onto T-HD can be predicted as the physical adsorption process.

The Langmuir, Freundlich, and D-R isotherm parameters were determined from the slopes and intercepts of the respective plots as summarized in Table 2. The experimental data fit well to the Freundlich isotherm with a correlation coefficient R^2^ of 0.994. According to Freundlich isotherm fitting, the value of 1/n is 0.885 (lower than 1.0), indicating favorable adsorption of Cu(II) on T-HD. The results showed strong positive evidence of the heterogeneous adsorption and indicated multilayer coverage of Cu(II) on the T-HD adsorbent. The interaction of Cu(II) and T-HD was also confirmed by the value of the E_a_ (0.270 J mol^-1^) of D-R, demonstrating physical adsorption at equilibrium.

**Table 2:** Isotherms parameters of Cu(II) adsorption on T-HD at contact time 20 h, pH 4, adsorbent dose 4 g L^-1^.

Langmuir			Freundlish			Dubinin-Radushkevich			
R^2^	K_L_	q_m_ (mg g^-1^)	R^2^	K_F_	1/n	R^2^	q_m_ (mg g^-1^)	K_D_ (mol^2^ J^-2^)	E (J mol^-1^)
0.746	1.12 × 10^–5^	2.07.10^3^	0.994	0.0595	0.885	0.908	375	6.85	0.270

To study the thermodynamics of the adsorption process, T-HD was immersed in Cu(II) aqueous solution at adsorbent dose of 4 g L^-1^, pH 4 at 10, 20, 30 and 40 °C and shaken at 120 rpm until equilibrium was reached. The thermodynamic parameters $\Delta \text{G}^\circ$, $\Delta \text{H}^\circ ,$ and $\Delta \text{S}^\circ$ were calculated by using the following equations.

13 $$\Delta G^\circ =-RTln{k}_{d}$$

The standard enthalpy ($\Delta \text{H}^\circ$) and entropy (ΔS$^\circ$) can be determined from the slope and intercept of the plot between $ln{k}_{d}$ and $\frac{1}{T}$ of the van’t Hoff equation, respectively.

14 $$ln {k}_{d} = \frac{\Delta S^\circ }{R}-\frac{\Delta H^\circ }{RT}$$

where $\Delta \text{G}^\circ$ (kJ mol^-1^) is the Gibbs free energy, $\Delta \text{H}^\circ$ is the enthalpy (kJ mol^-1^), $\Delta \text{S}^\circ$ (J mol^-1^ K^-1^) is the entropy, ${k}_{d}$ is the equilibrium constant, R is the gas constant, and T (K) is the solution temperature.

The thermodynamic parameters:$\Delta \text{H}$, $\Delta \text{S}$, and $\Delta \text{G}$, were used to evaluate whether the process is spontaneous. The Gibbs free energy change $\Delta \text{G}$ of the process listed in Table 3 were negative (-7.97, -8.17, -8.13, and -8.15 kJ mol^-1^), representing a spontaneous adsorption processes for Cu(II) onto T-HD at temperature 10, 20, 30 and 40 °C, respectively. The change in enthalpy $\Delta \text{H}$ was -6.592 kJ mol^-1^, which indicated exothermic adsorption. The change in entropy $\Delta \text{S}$ of 5.081 J K^-1^ mol^-1^ indicated increasing in randomness at the solid-solution interface during the adsorption of Cu(II) onto T-HD^53–55^. As shown in Table 4, The T-HD hydrogel exhibited superior adsorption properties for Cu(II) compared to other reported adsorbents. T-HD exhibited higher sorption capacity than other adsorbents. This higher performance was attributed to high adsorption capacity and efficient removal of Cu(II) in acidic conditions.

**Table 3:** Isotherms parameters of Cu(II) adsorption on T-HD at contact time 20 h, pH 4, adsorbent dose 4 g L^-1^.

T (K)	$\Delta \text{G}$(kJ mol^-1^)	$\Delta \text{H}$(kJ mol^-1^)	$\Delta \text{S}$(J mol^-1^ K^-1^)
283.15	-7.97 ± 0.07	-6.592	5.081
293.15	-8.17 ± 0.03		
303.15	-8.13 ± 0.18		
313.15	-8.15 ± 0.20

**Table 4:** Maximum adsorption capacity (mg g^-1^) of different adsorbents for Cu(II) adsorption from previous works.

Type of adsorbent	pH	Maximum adsorption capacity (mg g^-1^)	T (°C)	Isotherm fitting model	Kinetic fitting model	Reference
Natural clay	6	64.56	25	Freundlich	Second order	^56^
Walnut shell derived activated carbon	6	89.29	20	Langmuir	Pseudo-second order	^57^
Carbon aerogel	6	86.27	25	Langmuir	Pseudo-second order	^58^
AC-NaOH@PEI	6	47.8	25	Langmuir	Quasi second order	^59^
Poly(Acrylic acid/chestnut shell pigment) hydrogel	6	200.3	27	Langmuir	Pseudo-first order and pseudo-second order	^60^
Acrylic Acid-Chitosan Hydrogel	5.5	171	25	Langmuir	Pseudo-second order	^61^
CTS/CA/BT composite	5	115.3	25	Freundlich	Elovich	^62^
*Lagenaria vulgaris* shell	5	33.34	20	Langmuir	Pseudo-second order	^9^
Oxidized activated carbon	4	117.4	25	Langmuir	-	^37^
SS-CS@DTPA	3	31.42	25	BiLangmuir	Pseudo-second order	^63^
T-HD hydrogel	4	297.0	30	Freundlich	Pseudo-second order	This work

### Regeneration

**Fig. 5:**
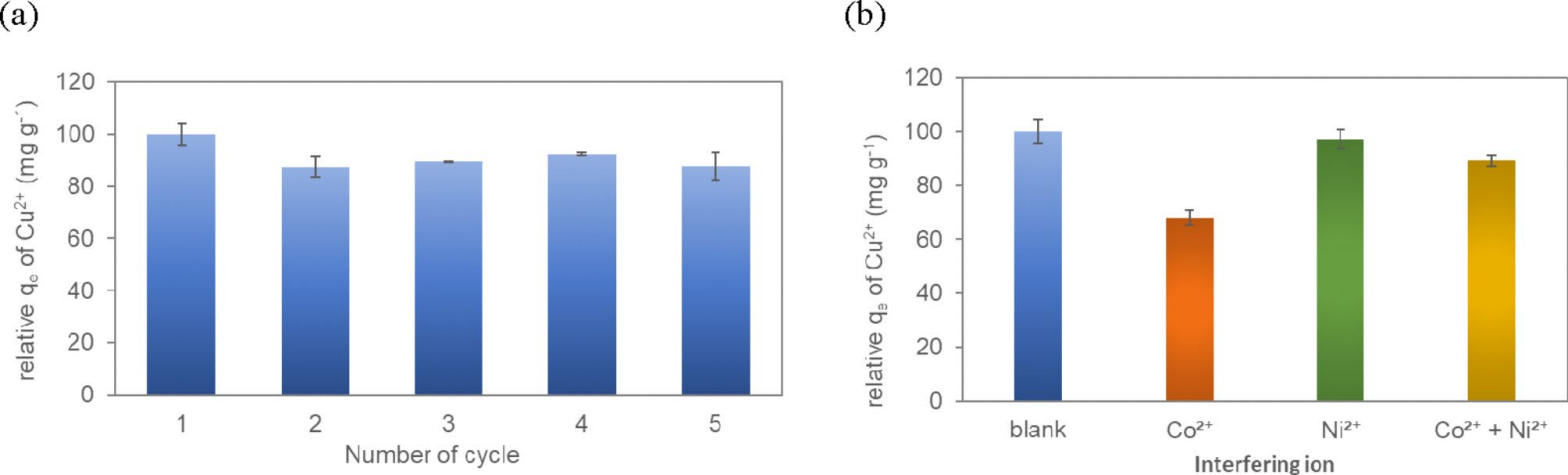
Relative q_e_ of Cu(II) for (**a**) Cycle regeneration (**b**) Interfering ion.

The cycle regeneration study was performed to evaluate the stability and reusability of T-HD^64,65^. Cu(II) can be desorbed washing Cu/T-HD with 0.1 M EDTA. The competition between Cu(II) forming complexes and EDTA led Cu(II) desorption similar to adsorption-desorption of metal ions onto chitosan monoliths^66^. The consecutive desorption-adsorption study showed that the adsorption efficiency of T-HD (0.10 M) decreased in cycles 2 to 5 to 87.4%, 89.3%, 92.2% and 87.8%, respectively (Fig. 5a). The results suggested that some quantities of Cu(II) ions might remain on the T-HD after adsorption, and the saturation of ions adsorbed onto the available adsorption sites would limit further adsorption. Based on the results, T-HD can be used as a metal ion adsorbent and still retained a good adsorption property without a significant decrease in the adsorption capacity after the fifth regeneration cycle. The results suggested that the cross-linking interaction between TA and HD films was strong enough to withstand dissolution for five regeneration cycles in acidic conditions, indicating a robust adsorption performance.

### Interfering ions

The influence of competing metal ions on the adsorption of Cu(II) was determined with competing metal ions at pH 4. The experiment was conducted to investigate the adsorption capacity of Cu(II) by T-HD in the presence of interfering ions including Ni(II) and Co(II) ions. In this study, selective Cu(II) adsorption with competing-metal ions was observed by using a simple UV-vis spectrophotometer. Figure 5b. shows the adsorption capacity of Cu(II) by T-HD in three different solutions. The amount of Cu(II) adsorbed was reduced when Co(II) ion was introduced to the solution, whereas Ni(II) ion slightly reduced the adsorption of Cu(II). The adsorption capacity of Cu(II) on T-HD decreased by about 34%, 14%, and 6% when Co(II), Co(II)-Ni(II), and Ni(II) were separately added, respectively. The results demonstrated that the presence of Co(II) ions had a greater effect on the adsorption capacity of Cu(II) on T-HD. The decrease in the adsorption capacity of Cu(II) was due to cobalt incorporating into the T-HD interface and forming Co-O, consequently blocking Cu(II) to interact with the active site. Compared to Co(II), introducing Ni(II) ions to the solution slightly decreased the adsorption capacity of Cu(II). Based on this study, the adsorption capacities for Cu(II) on T-HD decreased in the order of Co > Co–Ni > Ni. Therefore, T-HD could be used to remove copper from a solution containing nickel and cobalt at once.

### Characterizations

#### Scanning *electron* microscopy (SEM)

**Fig. 6:**
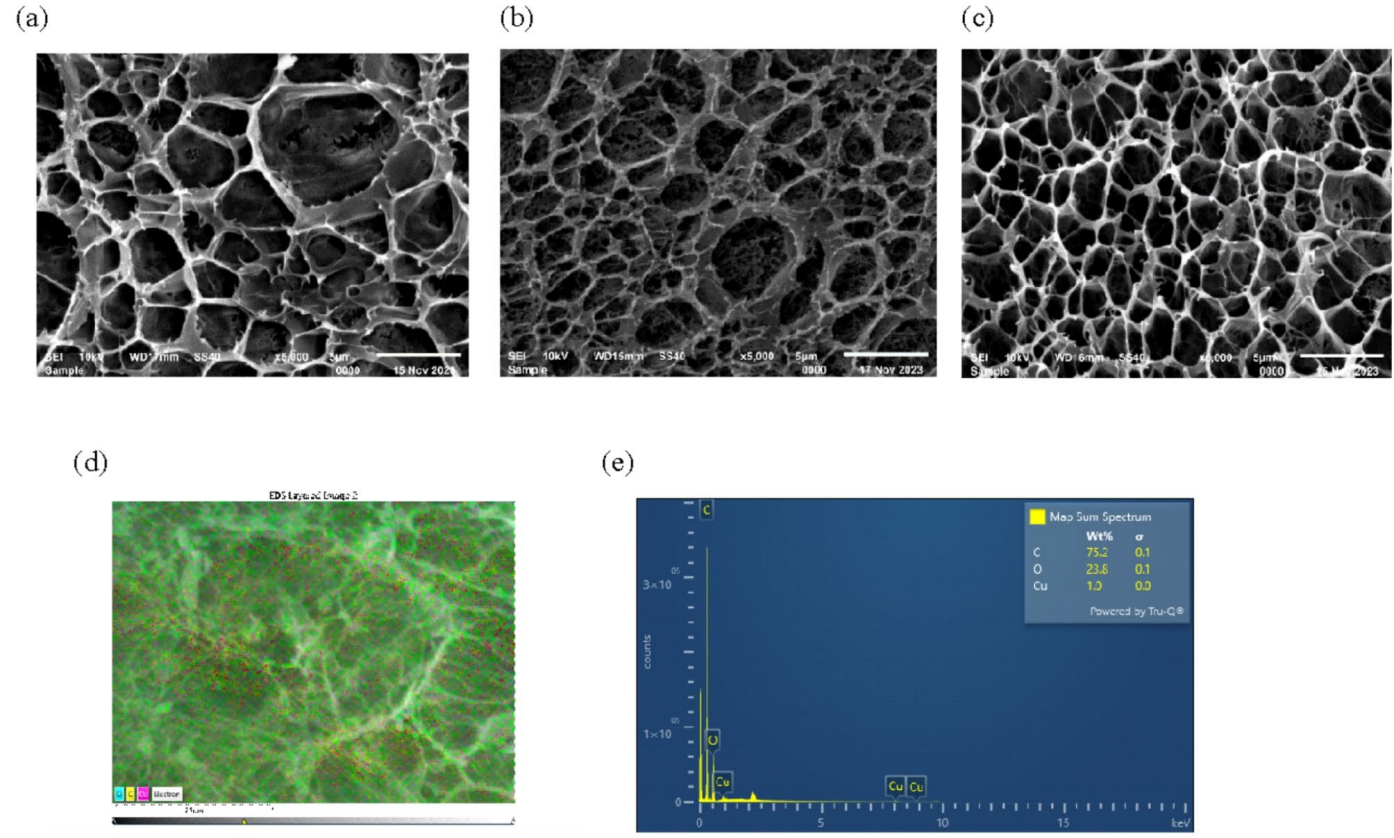
SEM images of hydrogel (**a**) T-HD (**b**) HD (**c**) Cu/T-HD (**d**-**e**) SEM/EDS of Cu/T-HD.

SEM was used to characterize the morphology of adsorbent materials. Figure 6a. revealed a microporous structure of the T-HD. Compared to the HD (2.97 ± 0.4 µm) (Fig. 6b.), the incorporation of tannic acid into the HD (2.81 ± 0.4 µm) reduced the pore size. The SEM images of T-HD showed a smooth internal three-dimensional microstructure of dense network. The crosslink density was improved by multiple interactions between phenolic groups and polymer chains from the addition of tannic acid into the hydrogel. The pores were interconnected, and a more porous structure can be constructed during the polymerization process^31,32^. Such microporous structure with high porosity in T-HD can facilitate the diffusion and accessibility of the active sites to adsorbate particles, thus exhibiting a great adsorption potential for the removal of Cu(II) from water.

After adsorption, the morphological difference was observed between adsorbed T-HD and T-HD adsorbents. The SEM-EDS images showed that Cu(II) aggregated on the surface of adsorbed T-HD adsorbent (Fig. 6d, e) with the average pore diameter of 2.09 ± 0.3 µm (Fig. 6c). This indicates that upon Cu(II) adsorption, active sites of T-HD interacted with Cu(II) ions and accumulated on the surface of the adsorbent, leading to a reduction in pore size.

From the BET model, the Langmuir surface area of the T-HD was 3.029 m^2^ g^-1^, comparable to that of other^38^ and the total pore volume was 0.00127–0.00163 cm^3^ g^-1^. The pore size distribution showed a mesoporous material, with an average pore diameter of about 14.6 nm. The surface of the adsorbent after Cu(II) adsorption became smaller mesopores with an average diameter of about 3.6 nm (Table S1). The pore volume of Cu/T-HD exhibited a tenfold increase compared to T-HD. This significant enhancement likely stemmed from the aeration of smaller mesopores and micropores during Cu(II) adsorption. This increase in pore volume, coupled with the substantially larger Langmuir surface area (274.6 m^2^ g^-1^), suggests that the modification significantly increased the amount of accessible surface area, potentially by introducing more fine pores^67,68^.

#### Fourier transform infrared spectroscopy (FTIR)

**Fig. 7:**
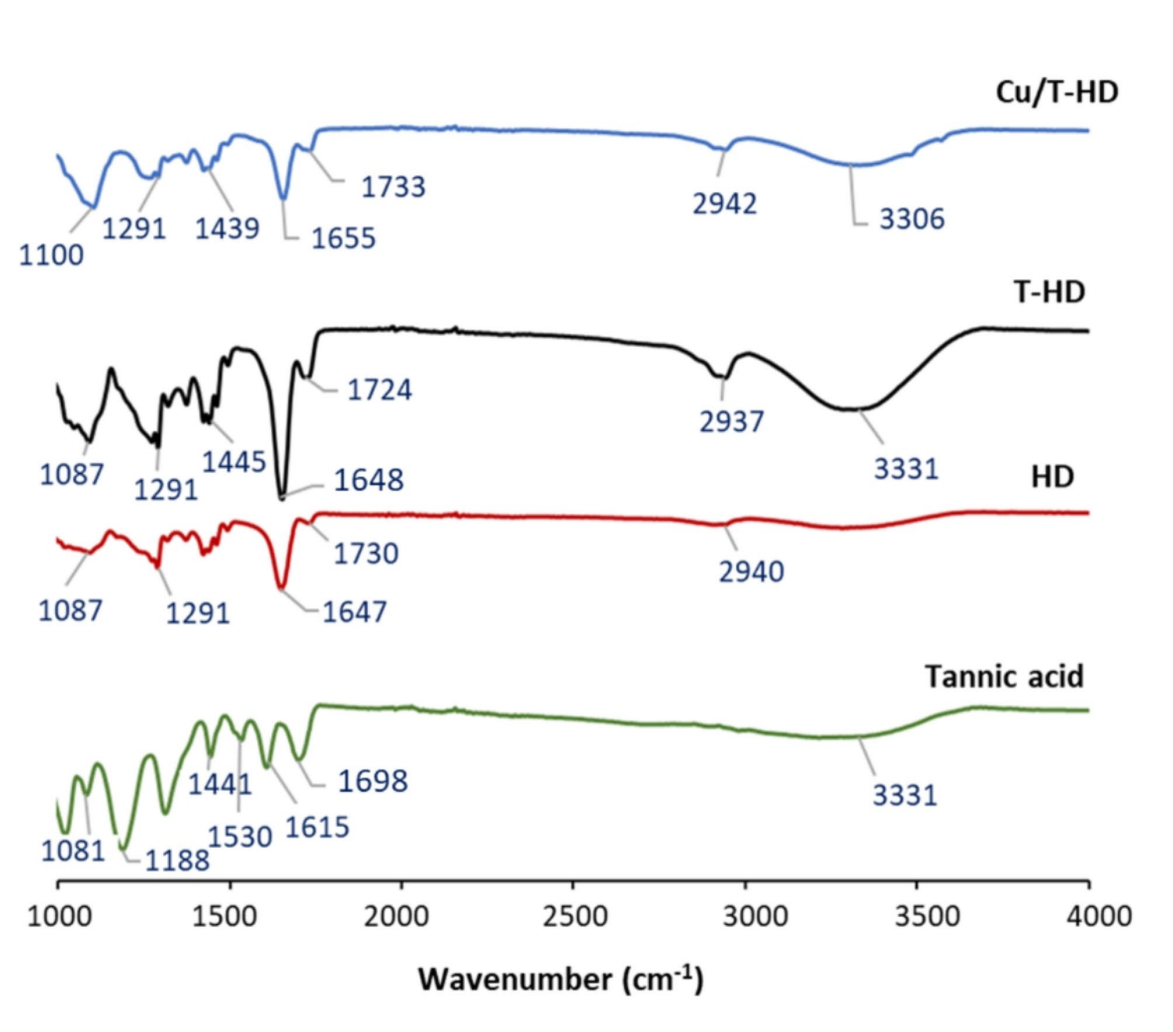
FTIR spectra of tannic acid, HD, T-HD, and Cu/T-HD.

FTIR was used to reveal the interactions between TA and polymers. Figure 7. presents the functional-group compositions of the TA, HD, T-HD, and Cu/T-HD characterized from FTIR spectra. In the spectrum of TA, the board absorption band around 3331 cm^-1^ and 1530 cm^-1^ corresponded to the stretching vibration of the phenolic (-OH) groups and aromatic C = C, respectively. A prominent peak at 1698 cm^-1^ was assigned to C = O stretching of the phenolic ester group in TA. For T-HD, the characteristic -OH stretching band of TA was stronger than that of the HD^69^. The C = O stretching frequency of carboxyl groups at 1730 cm^-1^ in HD was shifted to 1724 cm^-1^ in T-HD^25,70^. The shift of C = O vibration to the lower wavenumber was caused by the reduction of force constants of the chemical bonds resulting from the formation of hydrogen bonds^28,71^. These results confirm that the formation of hydrogen bonds occurred through -OH and C = O groups, suggesting that tannic acid was successfully crosslinked to the polymer.

The FTIR spectra before and after adsorption were compared to determine the interactions between Cu(II) and the functional groups of the adsorbents. After Cu(II) adsorption, the wide band of the -OH vibrations around 3331 cm^-1^ slightly decreased and shifted to the lower wavenumber at 3306 cm^-1^ corresponding to the reaction at the hydroxyl group^64,69^. Moreover, the peaks at 1724 and 1648 cm^-1^, assigned to C = O and NC = O units of T-HD, respectively, were offset and degenerated with the neighboring peaks. These changes were possibly due to bonds formed between hydroxyl, carbonyl, and carboxyl groups and the Cu(II) ion species, which affected bond strength and caused the vibrational band shifts^72^. The electrons present in the oxygen in the O–H, C = O, and NC = O groups can establish a coordinate bond with Cu(II) ions. The results of FTIR analyses proved that the hydroxyl and carbonyl groups on the T-HD surface were the main active sites involved in the Cu(II) adsorption process and effectively increased the adsorption capacity for heavy metal ions.

#### X-ray photoelectron spectroscopy (XPS)

**Fig. 8:**
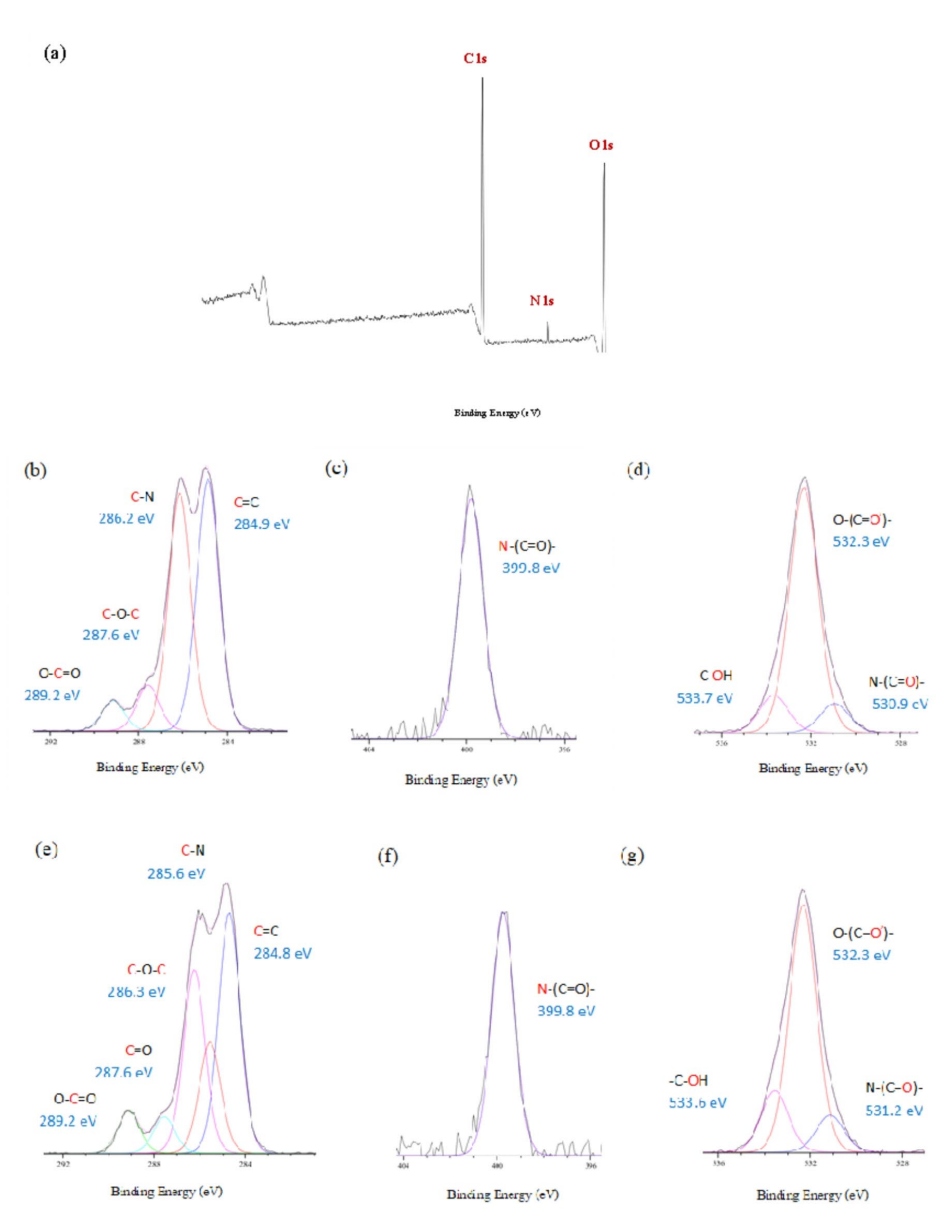
XPS survey spectra of T-HD (**a**) and high resolution of C1s, N1s and O1s of the T-HD (**b**), (**c**), (**d**) and Cu/T-HD (**e**), (**f**), (**g**).

XPS was used to investigate the chemical surface composition of the T-HD before and after metal adsorption. The XPS survey scan of bare hydrogel showed the presence of C1s, N1s, and O1s, with high intensity of both C1s and O1s peaks. As shown in Table S2., the percentage ratio of O/C of T-HD (0.56%) was higher than that of HD (0.48%). The results suggested that there was the enrichment of carbon oxidation surface functional groups on T-HD film, which dominated the metal adsorption. The greater oxygen-containing functional groups on the T-HD surface provided more electrons for metal to form bonds with T-HD during the adsorption. To further investigate, the high-resolution XPS spectra of T-HD before and after Cu(II) adsorption were recorded (Fig. 8). Before the adsorption, the C1s region of T-HD showed four component peaks at 289.2, 287.6, 286.2 and 284.9 eV, which were attributed to O-C = O, C-O-C, C-N and C-C/ C = C respectively^73^. The O1s spectra exhibited three peaks at 533.7, 532.3, and 530.9 eV were assigned to –C-OH, O-(C = O^′^)-, and N-(C = O)-, respectively. After the adsorption, the O1s spectra were deconvoluted into three peaks, identified as -C–OH at 533.6 eV, O-(C = O^′^)- at 532.3 eV and the N-(C = O)- at 531.2 eV. The increase in binding energy of O1s representing N-(C = O)- at 530.9 eV to 531.2 eV indicated that electron transfer occurred between O containing functional group on the surface and metal species^74^. Bonding compositions of T-HD after Cu(II) adsorptions are shown in Table S3. To confirm the chemical species of the functional groups on the T-HD with metal during metal adsorption, the XANES, and EXAFS were used for further studies.

#### X-ray absorption spectroscopy (XAS)

**Fig. 9:**
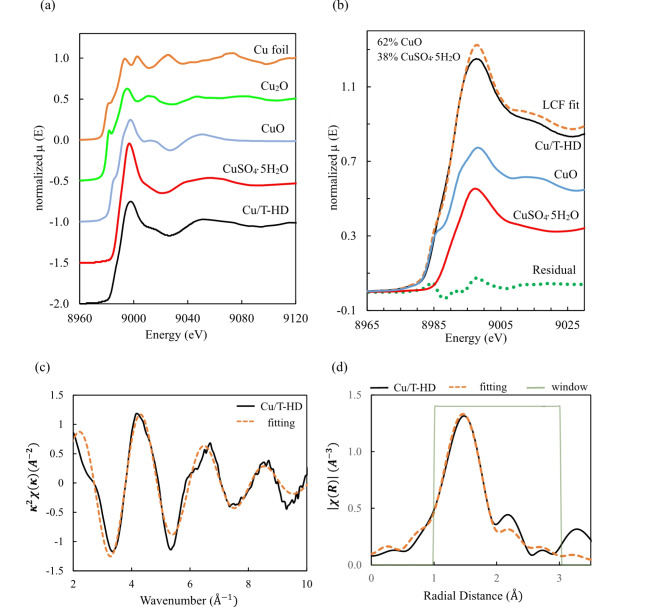
(**a**) Comparison of XANES Cu K-edge of Cu/T-HD and other Cu standards. (**b**) Chosen CuO and CuSO_4_⋅5H_2_O in the linear combination fitting to Cu/T-HD and their residual. (**c**) EXAFS fitting spectra in term of wavenumber and (**d**) radial distance.

XANES and EXAFS of Cu K-edge were used to investigate mechanism of Cu adsorption on T-HD without any competitive elements^75^. To elucidate the oxidation state of Cu on T-HD, XANES of Cu K-edge in Cu/T-HD system was plotted in comparison to other forms of Cu e.g. Cu-foil, Cu_2_O, CuO and CuSO_4_⋅5H_2_O. The spectra are shown in Fig. 9a. Linear combination fitting (LCF) of Cu/T-HD was performed with XANES of CuO and CuSO_4_⋅5H_2_O. LCF revealed that the Cu/T-HD had percentages of composition of the CuO and CuSO_4_⋅5H_2_O at 62 and 38%, respectively. The best fit against XANES of Cu/T-HD are shown in Fig. 9b. with residual between LCF and actual data shown in the green dotted line. EXAFS fitting of Cu/T-HD spectra was calculated. Structures of monoclinic CuO (mp-704645) and hydrated CuSO_4_ (mp-20525) were imported and the first shells of oxygen atoms in both structures were added to the calculation^75^. To reveal the local structure around copper atoms. The EXAFS fitting was conducted using single scattering paths with specific coordination numbers. In fitting, the amplitude reduction factor (*S*_0_^2^) was constrained within the range of 0.7 to 1.0. The Debye-Waller factor (σ^2^) was controlled to be 0.005 Å^2^, reflecting the low level of disorder in the system. The energy shift parameter (ΔE) was maintained below 10 eV which expresses acceptable inaccuracies in the edge energy determination. This fit achieved an R-factor of 1.8%, indicating a good agreement between the model and the experimental data. The detailed parameters utilized in the EXAFS fitting are presented in Table 5. In addition, the results illustrate the quality of the fit, with the $\kappa$ ^2^ χ(κ) and |χ (*R*)| are shown in Fig. 9c and d., respectively. These figures demonstrate the excellent agreement between the experimental and fitted data, thereby validating the robustness of the fitting procedure and the reliability of the extracted structure.

**Table 5:** EXAFS fitting parameters of Cu K-edge in Cu/T-HD system.

Compounds					
CuO (monoclinic) 62%		CuSO_4_⋅5H_2_O 38%			
Cu-O_1_		Cu-O_2_		Cu-O_3_	
R(Å)	σ^2^(Å^2^)	R(Å)	σ^2^(Å^2^)	R(Å)	σ^2^(Å^2^)
1.958 ± 0.052	0.005 ± 0.002	1.805 ± 0.216	0.005 ± 0.002	1.924 ± 0.216	0.005 ± 0.002

**Fig. 10:**
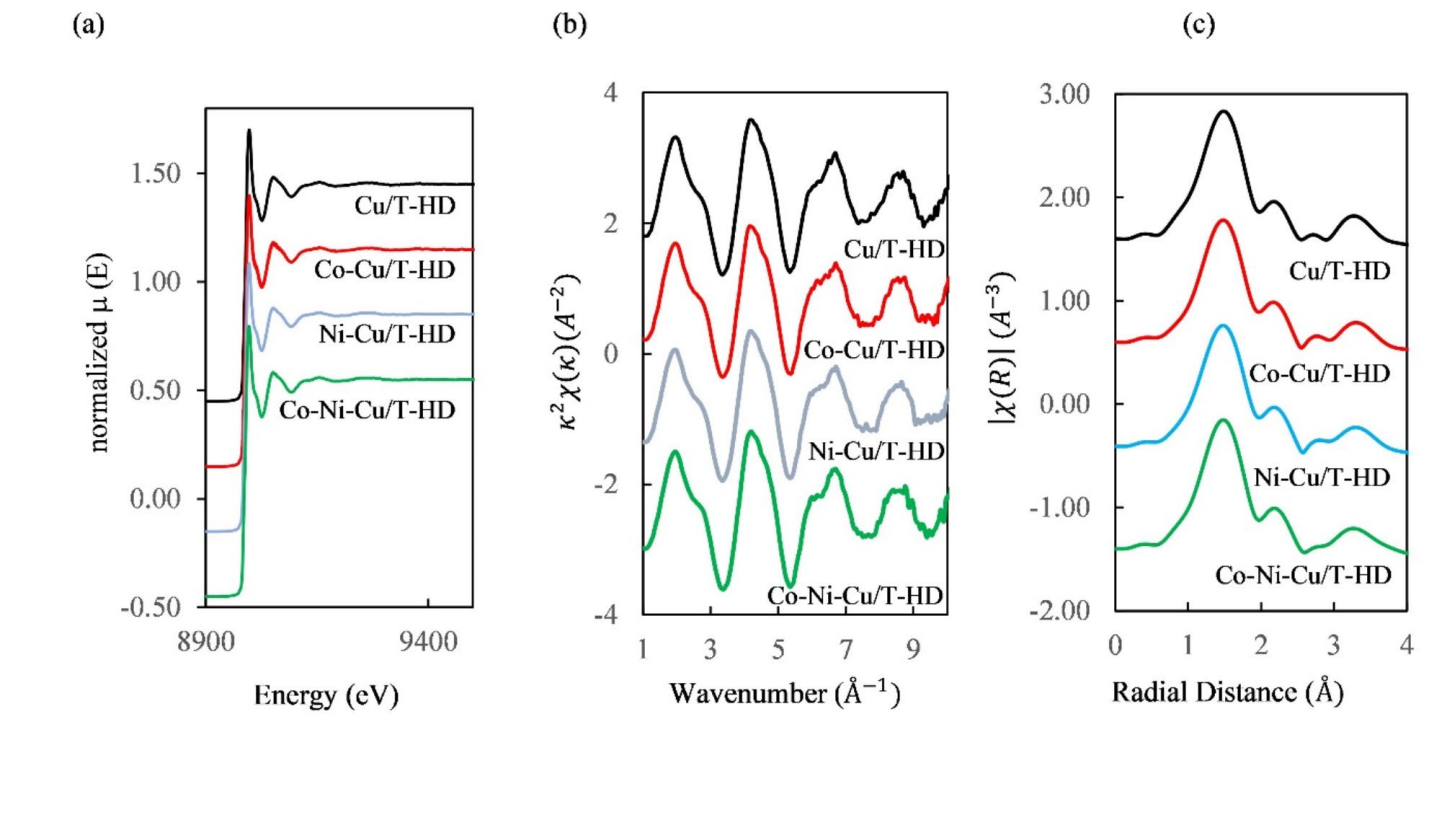
(**a**) XANES of Cu/T-HD, Co-Cu/T-HD, Ni-Cu/T-HD and Co-Ni-Cu/T-HD. (**b**) ${\kappa }^{2}\chi \left(\kappa \right)$ of Cu/T-HD, Co-Cu/T-HD, Ni-Cu/T-HD and Co-Ni-Cu/T-HD and (**c**) $\left|\chi (R)\right|$ of Cu/T-HD, Co-Cu/T-HD, Ni-Cu/T-HD and Co-Ni-Cu/T-HD.

To elucidate the influence of Ni and Co interfered on the system, the spectra of XANES and EXAFS of Cu K-edge when Cu absorbed on T-HD with Co, Ni, and both Co-Ni were measured. The spectra were plotted as shown in Fig. 10a. XANES of systems apart from that of Cu/T-HD exhibited similar features to that of Cu/T-HD even in ${\kappa }^{2}\chi \left(\kappa \right)$ (Fig. 10b) and $\left|\chi (R)\right|$ (Fig. 10c). Thus, this indicated that the co-absorption of Co, Ni and Co-Ni did not have an influence on the behavior of Cu absorbed to the T-HD. This behavior emphasizes the selective ability of the T-HD in the adsorption of copper. It has the selective ability even if there are Co, Ni or Co-Ni mixed ions contaminated in the system which can occur in the actual industrial situation. The T-HD still performs high efficiency in Cu adsorption.

#### Adsorption mechanism

**Fig. 11:**
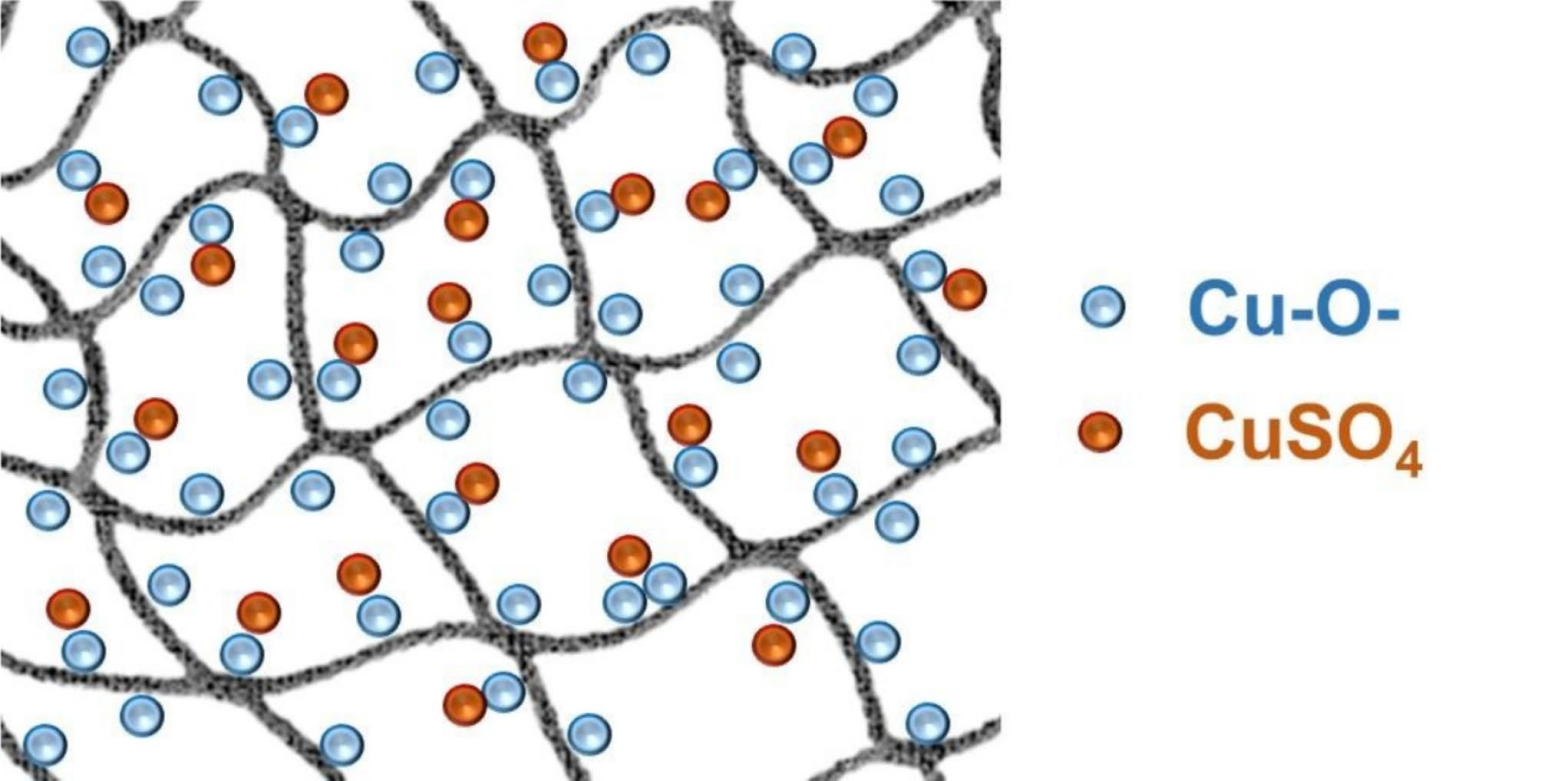
Reaction mechanism of the T-HD composite hydrogel.

To elucidate the complex adsorption mechanism, kinetics studies and isotherm models were used to analyze the adsorption data. This approach provided insights into the adsorption behavior, supporting the detailed understanding of the underlying mechanism. The adsorption behavior of Cu(II) by T-HD suggested an initial diffusion of particles into the pores of the adsorbent, contributing to both external diffusion and intraparticle diffusion process. After that, Cu(II) interacted with the oxygen-containing functional groups (-OH, C = O and N-(C = O)-) of T-HD to form complex adsorption at the T-HD surface. The kinetic study indicated that the adsorption behavior of Cu(II) by T-HD followed pseudo-second order kinetic. This means that the adsorption rate was controlled by the chemical interaction through an exchange of electrons between Cu(II) and the oxygen-containing functional groups. Subsequently, more Cu(II) particles were adsorbed and formed multilayer on the T-HD surface at the equilibrium adsorption. This phenomenon might explain why a high concentration of Cu(II) was used in the batch adsorption experiments. The adsorption mechanism of Cu(II) on T-HD is shown in Fig. 11.

## Conclusions

This work presents the development of tannic acid-incorporated polyvinylpyrrolidone/polyvinyl alcohol composite hydrogel (T-HD) as an effective adsorbent for Cu(II) removal from aqueous solutions. Compared with a bare hydrogel (HD), the modified hydrogel enhanced and reinforced functional groups to improve the adsorption capacity of the hydrogel for Cu(II). The results demonstrated the potential of T-HD as an adsorbent for Cu(II) removal due to its three-dimensional porosity and high affinity for the adsorption reaction. Under optimal conditions, the T-HD adsorbent could remove Cu(II) from water, with a maximum adsorption capacity (q_m_) of 297.0 mg g^-1^ at pH 4. Thermodynamic studies revealed that the adsorption process was spontaneous and exothermic, and was characterized by a positive change in the entropy value. The experimental data revealed that T-HD exhibited high adsorption performance for Cu(II) ions not only under single-solute conditions but also under coadsorption conditions in acidic aqueous solutions. The Cu(II) adsorption mechanism involves external diffusion and intraparticle diffusion processes, along with complexation of Cu(II) with oxygens in the -OH, C = O and N-(C = O)- functional groups on the T-HD. Characterization of the orientation and composition of T-HD revealed two molecular geometries for Cu(II) on the adsorbent surface. These geometries were identified as Cu(II) ions adjacent to oxygen, similar to those found in CuO and CuSO_4_, representing chemisorbed-copper(II) and physisorbed-copper(II), respectively. This finding highlighted the geometric arrangement differences between chemisorbed and physisorbed particles on the adsorbent surface. This understanding is crucial for comprehending the influence of surface composition on the performance of composite hydrogels in adsorption applications. The results of this study contribute valuable insights into the development of an effective adsorbent for the treatment of acidic wastewater containing high concentrations of metals discarded from electroplating industries.

## Electronic supplementary material

Below is the link to the electronic supplementary material.

Supplementary Material 1

Supplementary Material 2

## Data Availability

The datasets used and/or analyzed during the current study available from the corresponding author on reasonable request.
