# Supplementary material for: Fabrication of tannic acid-incorporated polyvinylpyrrolidone/polyvinyl alcohol composite hydrogel and its application as an adsorbent for copper ion removal
Source: Sci Rep. 2024 Nov 16;14:28259. doi: 10.1038/s41598-024-80024-x (PMC11569224; doi:10.1038/s41598-024-80024-x)
Supplement: Supplementary file 1 — Supplementary Material 1 [file 41598_2024_80024_MOESM1_ESM.docx]

Supplementary Information

**Fabrication of tannic acid-incorporated polyvinylpyrrolidone/polyvinyl alcohol composite hydrogel and its application as an adsorbent for copper ion removal**

Parichart Chunhakowit^1^, Yada Phabjanda^1^, Atchara Aunwisat^1^, Wutthikrai Busayaporn^2^, Kriangsak Songsrirote^1^, Pornpimol Prayongpan^1^*

^1^ Department of Chemistry, Faculty of Science, Srinakharinwirot University, Bangkok, 10110, Thailand

^2^ Synchrotron Light Research Institute, Nakhon Ratchasima, 30000, Thailand

* Corresponding Author: [pornpim@g.swu.ac.th](mailto:pornpim@g.swu.ac.th) (ORCID ID: 0000-0003-4385-9109)


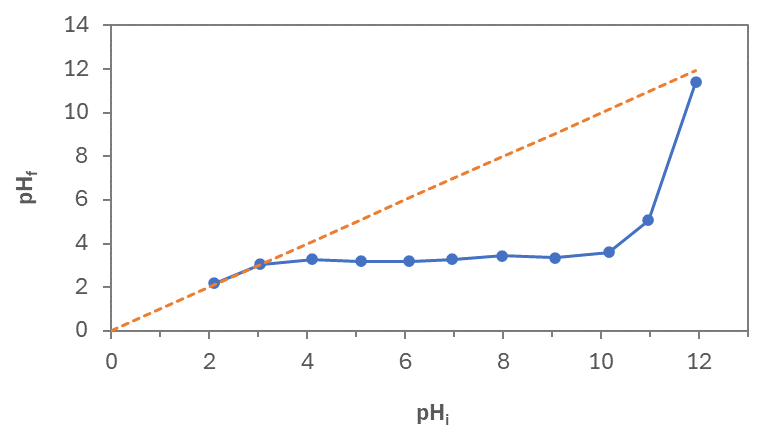


**Figure S1.** pH_pzc_ for T-HD.


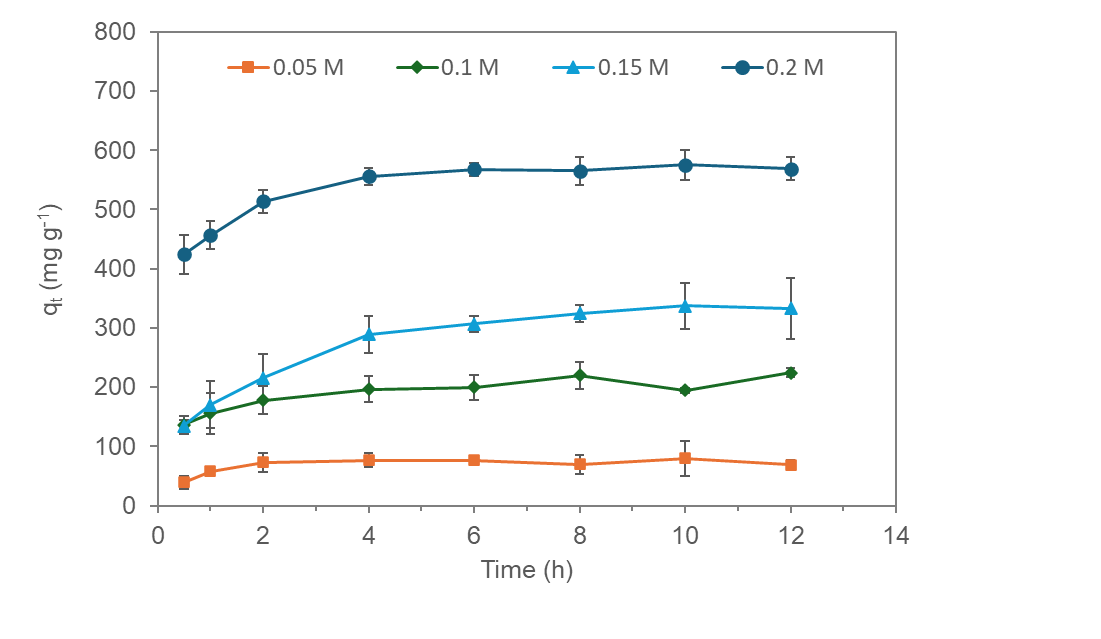


**Figure S2.** Effect of contact time (n=3) on the removal of Cu(II) by T-HD (initial concentration of 0.05−0.20 M, adsorbent dose 4 g L^-1^, temperature 30 °C).

**Table S1.** BET results of T-HD and Cu/T-HD.

| Material | Specific surface area  (m^2^ g^-1^) | Average pore diameter (nm) | Total pore volume  (cm g^-1^) |
| --- | --- | --- | --- |
| T-HD | 3.029 | 14.6 | 0.00127-0.00163 |
| Cu/T-HD | 274.6 | 3.6 | 0.01468-0.01734 |

**Table S2.** Elemental compositions of HD, T-HD, and Cu/T-HD.

| Composition | Elemental composition | | | | O/C ratio |
| --- | --- | --- | --- | --- | --- |
|  | %C | %N | %O | %Cu |  |
| HD | 62.36 | 5.01 | 30.24 | - | 0.48 |
| T-HD | 61.43 | 4.16 | 34.41 | - | 0.56 |
| Cu/T-HD | 60.85 | 3.29 | 35.32 | 0.55 | 0.58 |

**Table S3.** Bonding compositions of T-HD and Cu/T-HD.

| Material | | C1s | | | | | N1s | O1s | | |
| --- | --- | --- | --- | --- | --- | --- | --- | --- | --- | --- |
|  |  | C=C | C-N | C-O-C | C=O | O-C=O | N-(C=O)- | O-(C=O')- | C-O'H /  C-O'-C | O'-(C=O)- |
| T-HD | BE (eV) | 284.9 | 286.2 | 287.6 | - | 289.2 | 399.8 | 530.9 | 532.3 | 533.7 |
|  | Area (%) | 44.6 | 42.1 | 8.1 | - | 5.3 | 100 | 9.3 | 78.7 | 12 |
| Cu/T-HD | BE (eV) | 284.8 | 285.6 | 286.3 | 287.6 | 289.2 | 399.8 | 531.2 | 532.3 | 533.6 |
|  | Area (%) | 39.2 | 18.1 | 30 | 5.8 | 6.9 | 100 | 10.7 | 71.5 | 17.8 |
